# Effectiveness of moxibustion treatment as adjunctive therapy in osteoarthritis of the knee: a randomized, double-blinded, placebo-controlled clinical trial

**DOI:** 10.1186/ar4590

**Published:** 2014-06-24

**Authors:** Ling Zhao, Ke Cheng, Lizhen Wang, Fan Wu, Haiping Deng, Ming Tan, Lixing Lao, Xueyong Shen

**Affiliations:** 1 Shanghai University of Traditional Chinese Medicine, 1200 Cailun Road, Shanghai 201203, China; 2 Shanghai Research Center of Acupuncture & Meridian, 199 Guoshoujing Road, Shanghai 201203, China; 3 Department of Biostatistics, Bioinformatics and Biomathematics, Georgetown University Medical Center, 37th and O Streets, N.W., Washington, DC 20057, USA; 4 University of Maryland, School of Medicine, 655 West Baltimore Street, Baltimore, MD 21201, USA; 5 School of Chinese Medicine, University of Hong Kong, 10 Sassoon Road, Pokfulam, Hong Kong 999077, China

## Abstract

**Introduction:**

Our objective was to compare the effectiveness and safety of traditional Chinese moxibustion to that of sham moxibustion in patients with chronic knee osteoarthritis (KOA) pain.

**Methods:**

We conducted a randomized placebo-controlled trial involving 110 patients with KOA who met the inclusion criteria. These patients randomly received either active moxibustion (n = 55) or sham moxibustion control (n = 55) at acupoints Dubi (ST 35), extra-point Neixiyan (EX-LE 4), and an Ashi (tender) point three times a week for 6 weeks. Effects were evaluated with Western Ontario and McMaster Universities’ Osteoarthritis Index (WOMAC VA 3.1) criteria at the end of the course of treatment and 3, 12, and 24 weeks after the initial treatment.

**Results:**

The WOMAC pain scores showed greater improvement in the active treatment group than in control at weeks 3 (*P* = 0.012), 6 (*P* <0.001), 12 (*P* = 0.002), and 24 (*P* = 0.002) as did WOMAC physical function scores of the active treatment group at week 3 (*P* = 0.002), 6 (*P* = 0.015), and 12 (*P* <0.001) but not 24 (*P* = 0.058). Patients and practitioners were blinded successfully, and no significant adverse effects were found during the trial.

**Conclusions:**

A 6-week course of moxibustion seems to relieve pain effectively and improve function in patients with KOA for up to 18 weeks after the end of treatment. Moxibustion treatment appears to be safe, and the usefulness of the novel moxa device was validated.

**Trial registration:**

Current controlled trial: ISRCTN68475405. Registered 4 April 2014.

## Introduction

Knee osteoarthritis (KOA), common among the elderly, significantly affects patient quality of life because of pain and physical activity limitations [1,2]. Today, in Chinese who are at least 60 years old, the prevalence of symptomatic KOA is 19.4% [3]. Pharmacological therapies are often ineffective, and agents such as non-steroidal anti-inflammatory drugs can cause undesired side effects.

Moxibustion, a modality of traditional acupuncture, is a non-invasive procedure that involves burning moxa, the herb *Artemisia vulgaris*, on or above the skin at acupoints, warming them in order to alleviate symptoms [4]. Moxibustion has been practiced along with acupuncture in China for thousands of years. Widely used to treat various disorders [5-7], it is reported to be effective for arthritis and pain [8-12]. However, well-designed, randomized, placebo-controlled clinical trials (RCTs) of moxibustion are scarce. The purpose of the present study was to evaluate the effectiveness and safety of traditional moxibustion in treating KOA and to validate a sham moxibustion device.

## Methods

### Research design

This was a double-blinded RCT (n = 110). Eligible patients were randomly assigned to receive either active (n = 55) or sham moxibustion (n = 55) three times a week for 6 weeks. Participants and practitioners were blinded to the treatment assignments. Using the standard outcome instrument, Western Ontario and McMaster Universities’ Osteoarthritis Index (WOMAC) for pain and function scores, independent assessors unaware of treatment assignment performed outcome assessments at weeks 3, 6, 12, and 24 after the initial treatment.

### Participants and recruitment

Recruitment of patients started in August 2009; the last patient was screened and enrolled in 2011. The trial concluded in April 2012. The study was carried out at outpatient clinics in three traditional Chinese medicine hospitals in Shanghai. Participants were recruited by advertisements in local communities. The study protocol was approved by the Institutional Ethic Review Committee of Chinese Clinical Trials Registry based in Chengdu, China. This RCT was also registered in the Chinese Clinical Trials Registry (ChiCTR-TRC-11001408) on 6 July 2011.

Inclusion criteria were (1) male or female, age of at least 45 years, with KOA diagnosed according to American College of Rheumatology criteria [13], including radiographic evidence of at least one osteophyte at the tibiofemoral joint in one or both knees (Kellgren-Lawrence score 2 or 3); (2) pain score of at least 3 points on a 10-point visual analogue scale for most days during the previous month; and (3) willingness to sign the consent form and be randomly assigned to either a treatment or a placebo group.

Exclusion criteria were (1) presence of serious medical conditions that precluded participation in study; (2) intra-articular corticosteroid or hyaluronate injections, knee surgery, or use of topical capsaicin cream during the preceding 6 months; (3) previous experience with moxibustion; and (4) planned events such as knee replacement that would interfere with participation in all 24 weeks of the study.

### Procedures

After a brief telephone screening, patients were scheduled to an initial visit in which they read, understood, and signed an informed consent and underwent a brief rheumatologic examination by a physician. Diagnosis was determined by using previously taken x-rays or those taken onsite. Eligible patients were randomly assigned to active moxibustion or sham control and scheduled for baseline assessment and treatment. We had all necessary consent from any patients involved in the trial, including consent to participate in the trial.

### Random assignment

Random assignment was generated by using computer software. Allocation concealment was ensured with letter codes that disguised patient names and groups. Patients were recruited in cohorts of 10; each cohort at each site was randomly assigned to one of two groups by a computer-generated process.

### Blinding

The practitioners, acupuncturists with at least 5 years of training in acupuncture and moxibustion, were divided, assigned to perform either active moxibustion on Tuesdays, Thursdays, and Saturdays or sham moxibustion on Mondays, Wednesdays, and Fridays and were taught to use the relevant device, real or sham. To perform a treatment, the practitioner took a moxibustion device from a box labeled with the code matching that of the patient. Because the real and sham devices appeared to be identical, practitioners and patients were blinded to treatment assignment. Blinding success was validated at the end of the study.

Patients (n = 55 per group) were treated three times a week for 6 weeks. The Chinese version of the WOMAC osteoarthritis (OA) indexes, shown to be valid and reliable for evaluating KOA pain and dysfunction, were used at baseline and follow-up assessments during the 24-week study period [14]. All patients were allowed to maintain their baseline pain medications during the course of treatment.

### Moxibustion devices

#### Real moxibustion device

**Figure 1:**
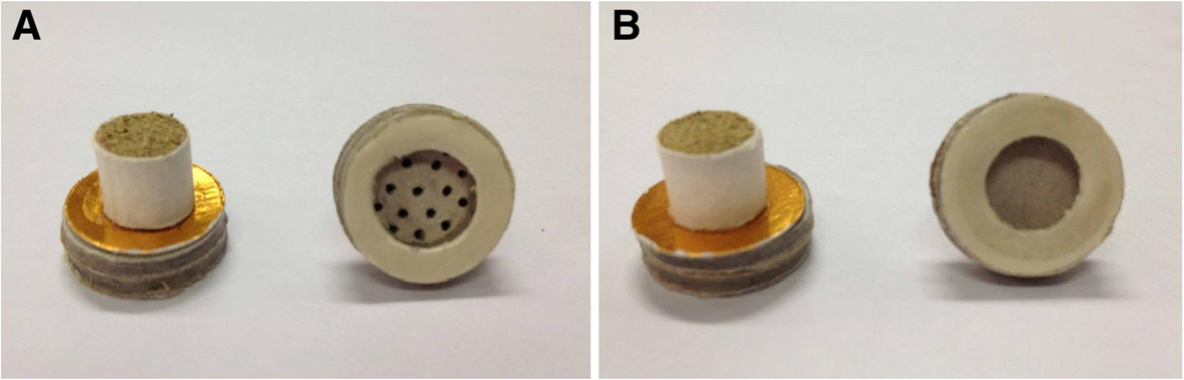
**Real and sham moxibustion pillar.** The real and sham moxibustion devices appear to be identical. **(A)** The real device has holes at the bottom to allow heat and smoke to radiate to the acupoints. **(B)** The sham device has a metal membrane at its base to block the smoke and minimize the heat.

We used a commercially available moxibustion device (Nanyang Hanyi Moxa Company, Ltd., Nanyang, Henan, China) (Figure 1). It has a cylindrical opening to hold a pillar of moxa; at its base is an adhesive membrane. During treatment, the device is placed at an acupoint, and the moxa is burned about 8 mm above the skin [15].

### Sham moxibustion device

The sham device resembles the real one in appearance, burning procedure, and residue after burning, but an insulated metal membrane over its base isolates the smoke and most of the heat, preventing them from radiating to the skin. Reliability of this device was previously tested and validated by Zhao and colleagues [15].

### Both devices are disposable

#### Acupoints and justification

The acupoints used, Dubi ST-35, extra-point Neixiyan (EX-LE4), and an Ashi (tender) point, are located in the knee area. These points are known to treat knee pain, including arthritic pain [16], and have been widely used in clinical trials [17-21].

#### Treatment procedure

The patient was supine during treatment. Patients in both groups were treated at three local points, ST 35, EX-LE4, and an Ashi point, in the area of the affected knee(s). Three consecutive moxa pillars were burned at each point. Once the device was affixed at a point, the first pillar was lit. After burning, the residual pillar was removed and another pillar was inserted and burned. A pillar burns for about 6 minutes, making the moxibustion session about 20 minutes long. The procedure was monitored by a research assistant to ensure that the device was not disassembled or checked by practitioners. After each treatment, the practitioner returned the box with burned moxa pillars to a box keeper, who was responsible for disposing of the used devices and unable to identify the patients treated.To maintain blinding, only naïve moxibustion patients were recruited, and the patients were told that they might or might not sense the heat from this newly developed device. As the active and sham devices look identical (Figure 1), patients and practitioners were all blinded.

### Outcome measures

Patients were assessed by using WOMAC index pain and function scores at weeks 3, 6, 12, and 24. The primary outcomes were the WOMAC pain and function scores taken at the end of the 6-week course of treatment. Secondary outcomes were WOMAC pain and function scores at weeks 3, 12, and 24. Two physicians blinded to treatment allocation performed the WOMAC assessments. If both knees were affected, the more severe knee was assessed. These methods have been previously reported [18,19,22-24].

Adverse effects were documented and evaluated with a standardized questionnaire developed in our previous studies [18,19]. Before each treatment, we assessed for possible adverse events, asking the patient, “Have you experienced any unusual symptoms since the last treatment?”

To validate practitioner blinding, each completed a questionnaire after every treatment to indicate which treatment they thought that they had administered. Patient blinding was assessed at the end of the 6-week course of treatment by using a previously validated questionnaire [19].

### Statistical considerations

The primary endpoint was the decrease of WOMAC score at week 6. Our sample size was calculated on the basis of previously reported RCTs of acupuncture versus sham control for knee OA [17,18] and our pilot study of moxibustion on knee OA [25]. We assumed a 36% decrease in WOMAC score to be effective, following the practice of others in OA [20]. The calculation equation we used was α = 0.05 and 1 - β = 0.90. Using STPLAN software, we determined that a sample size of 41 patients in each group would be sufficient to detect the statistical difference between the two groups; we enrolled 55 each to allow for a possible 20% dropout.

We calculated the percentage of change in WOMAC score at each given follow-up time from baseline as (post-treatment - baseline)/baseline × 100%. We used the intent-to-treat approach. The primary analysis was 6-week post-treatment improvement. Histograms and Q-Q plots were used to assess the distribution of the percentage changes. Wilcoxon rank-sum test or a two-sample *t* test was used, when appropriate, to test whether percentage changes in WOMAC scores were statistically different between treatment and placebo; the test level was α = 0.05. Fisher exact probabilities were used to analyze the dropout rate, patients’ drug use, and validation of patient blinding. Unadjusted *P* values for different tests were reported. We first determined, as a secondary analysis, whether the sphericity assumption in the repeated measures model would hold in order to provide an estimate of overall treatment effect throughout the time course [24]. As the assumption was not satisfied, the overall estimate was not appropriate to describe the treatment effect. Thus, individual estimates of the WOMAC scores at the various time points were reported.

## Results

**Figure 2:**
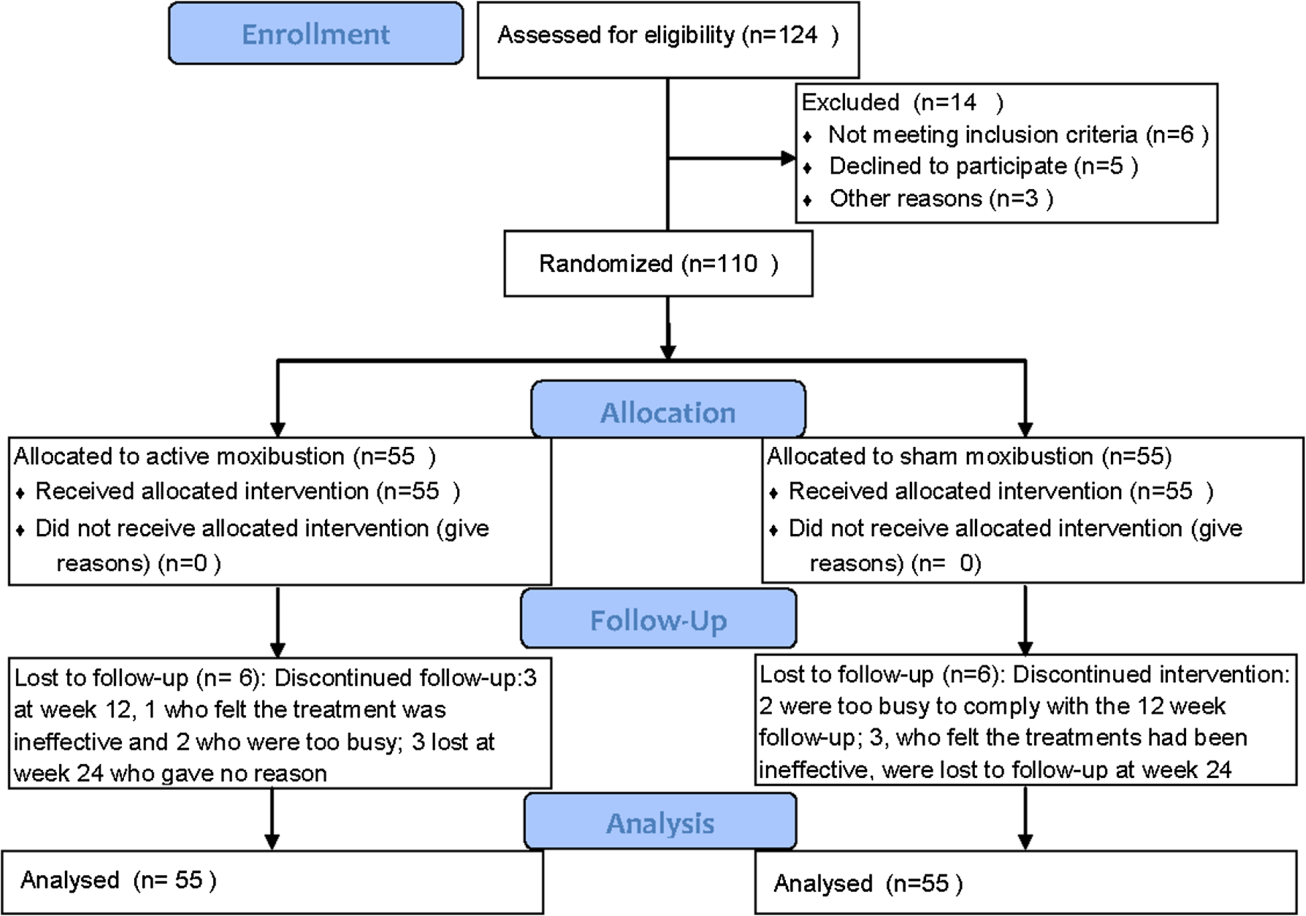
Flow diagram of the progress through the phases of the RCT.

In total, 124 eligible patients were screened and enrolled between August 2009 and October 2011; 14 were excluded: 6 because of insufficient pain, 4 for other clinically significant diseases, and 4 because they showed no radiographic evidence of an osteophyte. Of the 110 randomly assigned patients, all completed the 6-week course of treatment and 105 were assessed at 12 weeks (52 from the active group and 53 from the sham group). A total of 99, 49 from the active group and 50 from sham, completed the full 24 weeks (Figure 2). There was no difference between the two groups in age, gender, or course and severity of disease (*P* >0.05). Before treatment, there was no difference in WOMAC scores for knee pain or physical function between the groups (*P* >0.05). Baseline characteristics of the patients are presented in Table 1.

**Table 1:** **Participant demographic and baseline characteristics **$\bar{x}\pm s$

**Characteristics**	**Active group**	**Sham group**	**Total**
	**(n = 55)**	**(n = 55)**	**(n = 110)**
Age, years	65.80 ± 7.45	64.55 ± 8.38	65.17 ± 7.89
Gender, n (%)			
Male	16 (29.09)	21 (38.18)	37 (33.63)
Female	39 (70.90)	34 (61.81)	73 (66.36)
Target knees, n (%)			
1 knee	12 (21.82)	18 (32.73)	30 (27.27)
Both knees	43 (78.18)	37 (67.27)	80 (72.72)
Length of osteoarthritis diagnosis, n (%)			
<5 years	30 (54.55)	37 (67.27)	67 (60.90)
6-10 years	18 (32.73)	12 (21.82)	30 (27.27)
>10 years	7 (12.73)	6 (1.090)	13 (11.82)
Weight, kg	64.06 ± 9.02	66.01 ± 5.21	65.04 ± 6.33
Height, cm	1.63 ± 5.28	1.62 ± 1.45	1.62 ± 7. 98
Body mass index	24.11 ± 1.08	25.15 ± 2.41	24.63 ± 5.52
Outcomes (baseline)			
WOMAC pain score	6.73 ± 2.35	6.29 ± 2.70	6.51 ± 2.53
WOMAC function score	33.47.0 ± 15.37	30.99 ± 17.82	32.23 ± 16.61

There were no differences between the groups in age, gender, course of disease, or condition of the diseased knee (*P* >0.05). There were no differences between the groups in Western Ontario and McMaster Universities’ Osteoarthritis Index (WOMAC) pain or physical function scores (*P* >0.05).

### Pain

In all post-baseline assessments, WOMAC pain scores improved more in patients receiving active moxibustion than in those receiving sham (Table 2). Pain score at baseline was 6.69 ± 2.41 in the active group and decreased to 4.80 ± 2.47 at week 3; pain score at baseline was 6.27 ± 2.72 in the sham group and decreased to 5.56 ± 3.09 at week 3. The active group improved more (*P* = 0.012). By week 6, the primary outcome, mean WOMAC pain score, had significantly decreased to 3.03 ± 2.33 in the active group compared with 4.56 ± 3.09 in the sham group (*P* <0.001). At week 12, the active group decreased to 2.85 ± 2.67 versus 4.41 ± 3.65 in the sham group (*P* = 0.001). The differences remained significant through the last follow-up at week 24 (3.14 ± 2.42 versus 4.51 ± 3.29; *P* = 0.002).

**Table 2:** Comparison of WOMAC index score change (percentage) from baseline

**Endpoint**	**Week**	**Active group n = 55; (%)**		**Sham group n = 55; (%)**		**Z value**	***P *** **value**
		$\bar{x}\pm \mathrm{SD}$	**Median (P25, P75)**	$\bar{x}\pm \mathrm{SD}$	**Median (P25, P75)**		
Pain	3	24.65 ± 43.42	32.56 (9.74, 51.91)	2.63 ± 57.87	3.61 (-19.23, 44.44)	-2.513	0.012^a^
	6	52.87 ± 31.57	60.50 (33.70, 77.70)	24.43 ± 50.03	29.38 (3.28, 59.21)	-3.806	<0.001^a^
	12	57.90 ± 31.77	59.77 (38.46, 86.08)	18.37 ± 62.38	30.12 (-2.78, 65.15)	-3.052	0.001^a^
	24	50.75 ± 35.03	61.31 (31.08, 74.73)	20.47 ± 57.30	39.86 (-5.71, 75.00)	-3.111	0.002^a^
Function	3	21.71 ± 79.25	40.76 (9.89, 61.87)	-7.23 ± 96.90	15.93 (-11.44, 34.81)	-3.080	0.002^a^
	6	39.03 ± 71.26	54.37 (30.52, 73.65)	13.14 ± 111.49	34.50 (4.57, 65.97)	-2.423	0.015^a^
	12	50.84 ± 43.67	60.61 (32.82, 78.83)	14.51 ± 89.63	32.68 (-5.5, 64.20)	-3.543	<0.001^a^
	24	43.58 ± 53.51	58.32 (23.32, 74.11)	11.28 ± 114.82	39.86 (-5.71, 75.00)	-1.898	0.058

Western Ontario and McMaster Universities’ Osteoarthritis Index (WOMAC) score reduction change (post-treatment - baseline)/baseline × 100%. ^a^*P* <0.05 for purpose of comparison. SD, standard deviation.

### Physical function score

Physical function score in the active group had improved significantly compared with sham at week 3. Physical function score at baseline was 33.4 ± 15.37 in the active group and decreased to 22.10 ± 14.34 at week 3; physical function score at baseline was 30.99 ± 17.82 in the sham group and decreased to 26.71 ± 15.60 at week 3 (*P* = 0.002). By week 6, the active group was 16.43 ± 12.16 versus 21.70 ± 16.53 in the sham group (*P* = 0.015). At week 12, the active group was 14.61 ± 12.66 versus 21.98 ± 17.94 in the sham group (*P* <0.001). However, there was no significant difference between the groups at week 24 (15.92 ± 12.73 versus 20.50 ± 17.86; *P* = 0.058) (Table 2).

### Blinding

At the end of the trial, we asked practitioners to guess which group they believed they had been assigned to: three of the nine in the active group correctly guessed their group assignment, four guessed incorrectly, and one was uncertain; four of the nine practitioners in the sham group guessed correctly, three guessed incorrectly, and two were uncertain. The kappa consistency test (K = -0.53, *P* = 0.833) showed that the practitioners had been properly blinded.

Blinding effectiveness was assessed in patients at the end of week 6: 36% of the 110 patients correctly guessed their group assignment (18 out of 55 in the active group and 22 out of 55 in the sham control group), 32% guessed incorrectly (20 out of 55 in the active group and 15 out of 55 in the sham control group), and 32% were uncertain (17 out of 55 in the active group and 18 out of 55 in the sham control group). Fisher exact test showed no difference between the two groups in patient judgments (*P* = 0.565), showing that blinding was successful.

### Dropout rate analysis

The dropout rate was low; by the end of the trial, it was 10% (11 out of 110). In the active moxibustion group, six of the 55 patients were lost to follow-up: three at week 12; one felt the treatment was ineffective, and two were too busy to comply with the follow-up assessment; three at week 24 gave no reason. In the sham group, five of the 55 patients were lost to follow-up: two were too busy to comply with the 12 week follow-up; three, who felt the treatments had been ineffective, were lost to follow-up at week 24 (Figure 2).

### Adverse events

In the active moxibustion group, 10 patients reported skin flushing of about 5 mm in diameter at treatment sites after moxibustion. The flushing disappeared within 3 days without medical care. No other adverse event was observed as a result of treatment.

## Discussion

In this double-blind RCT, a 6-week course of moxibustion treatment significantly reduced pain and improved function in patients with KOA compared with sham control. Although the effectiveness of moxibustion treatment has been reported by others, the results of most of those studies were inclusive: the studies were of low quality and had high risks of bias [26,27]. In a systematic review on moxibustion for KOA, Choi and colleagues [27] found that the modality might be effective for symptom management in patients with KOA: 83% of the studies reviewed (39 out of 47) reported an effective rate. However, owing to the limited number of studies, their poor quality, and their inadequate use of controls and high risk of bias, the authors concluded that these RCTs provided little supporting evidence for their use in KOA [27].

In contrast, in the present trial, we addressed such limitations by using rigorous double-blinded, placebo-controlled methods with adequate random assignment. Another strength of our study is that the compliance rate in the first 6 weeks of the trial was high (100%), possibly because most of the participants were elderly and retired, with more time for treatment than those still working. Additionally, they all lived close to our hospitals, and many of them said that they found the treatment and the setting comfortable. Our trial is characterized as an effectiveness study, as defined by Gartlehner and colleagues [28]. The trial was conducted in a primary-care setting, the outcome was a condition-relevant health outcome, follow-up time was sufficient, and an intention-to-treatment statistical analysis was used.

To minimize the risk of bias, we adopted a novel sham moxibustion device first reported by Zhao and colleagues [15] in 2006. Similar sham moxibustion methods have been reported by several other investigators [29,30]. The uniqueness of our trial is the validated double-blinding. The nature of the moxibustion procedure makes blinding difficult, as patients might expect warmth to radiate from the burning moxa. In our trial, blinding was successful in part because all of the patients were naïve to moxibustion treatment. Additionally, appearance, burning process, and residue were the same in the sham and real devices; the only difference was the insulated metal membrane in the base of the sham device that minimized the moxa-produced heat and smoke.

The sham control device did produce some warmth but to a lesser extent than would true moxibustion. When we measured local skin temperature after treatment by each device, we found that active moxibustion produced 49.8°C on the skin versus the 40.9°C heat produced by sham [25]. Kim and colleagues [30] applied a sham device which was adjusted to the lowest possible temperature but which was enough to elicit a heat sensation of 39°C; the verum device was set at 44°C.

The fact that those receiving real moxibustion felt more warmth than did those given sham might has been a limitation, but we are confident that the patients were successfully blinded. Not only were the patients naïve to moxibustion, having never before experienced the procedure, the device also is unique. Patients were told that this was a newly developed device to test the effects of different temperatures of moxibustion, which enhanced the masking. Because the patients in the two groups were treated on different days, they were unable to communicate with each other about their treatment experiences. Similarly, the practitioners were assigned to either active or sham treatment; they had no chance to compare the two devices, obtained feedback only from patients of the same group, and were instructed not to discuss the patients’ feelings during treatment. We validated the effectiveness of patient blinding with a questionnaire; our data show that patients were effectively blinded to the treatment assignment.

The mechanisms of action of moxibustion therapy are still largely unknown. Factors such as temperature, smoke, odor, and herbs are likely to be involved in the possible mechanisms by which moxibustion may work [31]. A study showed that the effects of moxa sticks are related to the sensation of heat [32]. Moxibustion treatment is similar to acupuncture in principle, but in the former, the surface of the skin is stimulated with heat at acupoints; acupuncture treatment is widely known as “Zhen Jiu” (acupuncture and moxibustion) in the Chinese literature [4]. There is mounting evidence showing that acupuncture relieves pain and improves function in KOA [19,33-36]. Moxibustion might play a role similar to that of acupuncture stimulation, although its effect on the sensory nerve would be more superficial. Thermal stimulation might activate the sensory nervous system through peripheral nerves such as C fibers and A delta fibers, in turn transmitting sensory input to the central nerve system, which activates neurons to release beta endorphins and other neurotransmitters. Meanwhile, the afferent sensory input triggers the descending inhibitory pathway to the spinal level to intercept the pain signal. Heat at acupoints might also have local effects, dilating local blood vessels and increasing blood circulation [37]. A study showed that moxibustion stimulation is a reflex response; its afferent pathway is composed of somatic afferent nerves, and its efferent pathway involves the intracerebral cholinergic nerve [38].

There have been reports that degranulation of local mast cells and heat activation of thermoreceptors are possible mechanisms of moxibustion action [39]. Uryu and colleagues [40] observed the analgesic effects of moxibustion on an experimental KOA rat model and found that repeated moxibustion treatments for pain relief correlated with sustained inhibitory modulation by endogenous opioids.

## Conclusions

The findings of the present trial show that moxibustion, like acupuncture, can be a useful adjunctive treatment for patients with KOA. Moxibustion treatment is simple, easy to perform, and cost-effective. This modality is also more easily replicable than acupuncture, which is subject to variation caused by the different needling techniques of individual practitioners. Our findings suggest that traditional moxibustion is a safe, effective, and easy-to-use therapy that can be a useful adjunct to conventional medicine for alleviating pain and improving function in patients with KOA. A larger RCT using this double-blinded, placebo-controlled, multi-centered approach is warranted to confirm and generalize our findings.

## Abbreviations

KOA: knee osteoarthritis; OA: osteoarthritis; RCT: randomized controlled trial; WOMAC: Western Ontario and McMaster Universities’ Osteoarthritis Index.

## Competing interests

The authors declare that they have no competing interests.

## Authors’ contributions

LZ carried out the data collection and analysis, participated in the trial coordination, and drafted the manuscript. KC carried out the random assignment, allocation, and trial registration and drafted the manuscript. LW participated in trial coordination. FW and HD participated in data collection. MT carried out the data analysis. LL participated in the trial design and critically revised the manuscript. XS conceived of the trial and participated in its design and helped to draft the manuscript. All authors read and approved the final manuscript.

## Authors’ information

LZ and KC should be considered co-first authors.
